# Review of the protist *Labyrinhula* spp. and its relationship to seagrass disease under the influence of anthropogenic activities

**DOI:** 10.3389/fmicb.2024.1410195

**Published:** 2024-07-31

**Authors:** Qiuzhen Wang, Xinping Yu, Yike He, Yong Zhang, Ruixue Hui, Huike Ye, Caili Wang, Mohan Bai

**Affiliations:** ^1^Ocean College, Hebei Agricultural University, Qinhuangdao, China; ^2^Hebei Key Laboratory of Nutrition Regulation and Disease Control for Aquaculture, Qinhuangdao, China; ^3^Marine Geological Resources Survey Center of Hebei Province, Qinhuangdao, China; ^4^Ocean Survey Department, Qinhuangdao Marine Center of the Ministry of Natural Resources, Qinhuangdao, China; ^5^Agro-Environmental Protection Institute, Ministry of Agriculture and Rural Affairs, Tianjin, China; ^6^College of Life Sciences, Dezhou University, Dezhou, China

**Keywords:** *Labyrinthula*, protist, thraustochytrid, seagrass disease, human activity, climate warming, ocean acidification, blue carbon

## Abstract

Anthropogenic activities are driving significant changes in coastal ecological environments, increasingly spotlighting microorganisms associated with seagrass bed ecosystems. *Labyrinthula* is primarily recognized as a saprophytic protist associated with marine detritus, and it also acts as an opportunistic pathogen affecting marine algae, terrestrial plants and mollusks, especially in coastal environments. The genus plays a key role in the decomposition of marine detritus, facilitated by its interactions with diatoms and through the utilization of a diverse array of carbohydrate-active enzymes to decompose seagrass cell walls. However, human activities have significantly influenced the prevalence and severity of seagrass wasting disease (SWD) through factors such as climate warming, increased salinity and ocean acidification. The rise in temperature and salinity, exacerbated by human-induced climate change, has been shown to increase the susceptibility of seagrass to *Labyrinthula*, highlighting the adaptability of pathogen to environmental stressors. Moreover, the role of seagrass in regulating pathogen load and their immune response to *Labyrinthula* underscore the complex dynamics within these marine ecosystems. Importantly, the genotype diversity of seagrass hosts, environmental stress factors and the presence of marine organisms such as oysters, can influence the interaction mechanisms between seagrass and *Labyrinthula*. Besides, these organisms have the potential to both mitigate and facilitate pathogen transmission. The complexity of these interactions and their impacts driven by human activities calls for the development of comprehensive multi-factor models to better understand and manage the conservation and restoration of seagrass beds.

## 1 Introduction

Labyrinthulomycetes are a group of marine unicellular heterotrophic protists belonging to the Stramenopiles and the phylum Labyrinthulomycota, primarily consisting of thraustochytrids and labyrinthulids (Raghukumar, 2002; Table 1). The distinctive features of them are cell walls composed of sulfated polysaccharides and an ectoplasmic net structure formed by extensions from the cell membrane (Tsui et al., 2009). Thraustochytrid cells range from spherical to oval in shape, with the ectoplasmic net distributed on one side of the body and reproducing through the production of biflagellate zoospores of varying lengths (Moss, 1985). While labyrinthulid cells are spindle-shaped, covered externally by an ectoplasmic net and glide using this net (Moss, 1985; Popova et al., 2020). Thraustochytrids, inhibited by antimicrobial substances secreted by marine plants, are seldom found on living seaweeds and seagrass. Yet, they can parasitize certain invertebrates such as octopuses (Polglase, 1980), clams (Dahl et al., 2008) and squids (Jones and O'Dor, 1983), potentially causing infectious and lethal lesions directly or indirectly to the hosts. *Labyrinthula* is the only genus of labyrinthulids, and is a renowned protozoan pathogen, commonly existing in a parasitic or symbiotic manner inside living seagrass (Bergmann et al., 2011), marine algae (Popova et al., 2020), coral mucus (Burge et al., 2012) and terrestrial plants (Chitrampalam et al., 2015), leading to lethal lesions in these hosts. Furthermore, both groups of microbes are abundantly present on dead marine algae and decomposing mangrove leaves. They play a crucial role in the marine carbon cycle by degrading these rotting plants through the production of extracellular enzymes (Raghukumar, 2002).

**Table 1:** Compared characteristics of labyrinthulids and its closest relative thraustochytrids.

Species	Thraustochytrids	Labyrinthulids	References
Habitats	Marine	Marine, terrestrial	Bigelow et al. (2005) and Wang et al. (2019)
Colony color	Whitish or light pink	Whitish or colorless	Wang et al. (2019)
Zoospore	Yes	No	Moss (1985), Bigelow et al. (2005), and Popova et al. (2020)
Zoospore cell shape	Spherical	No	Wang et al. (2019)
Zoospore wall	Thick	No	Wang et al. (2019)
Trophic cell shape	Globose or sub-globose	Spindle, oblong to fusiform	Wang et al. (2019) and Popova et al. (2020)
Cell wall composition	Galactose, xylose, mannose and glucose	Fucose, galaetose and xylose	Chamberlain and Moss (1988)
Nutrition mode	Saprophytic, parasitic	Saprophytic, parasitic	Hassett and Gradinger (2018), Iwata and Honda (2018), Hamamoto and Honda (2019), and Popova et al. (2020)
Reproductive mode	Vegetative, amoeboid, sexual	No	Morabito et al. (2019)
Pathogenicity	Yes	Yes	Rubin et al. (2014) and Lee et al. (2021)
Host	Marine molluscs, Diatoms	Turf grass, seagrass, diatoms	Bigelow et al. (2005), Yoshioka et al. (2019), Hassett (2020), and Popova et al. (2020)
PUFA synthesis	Yes	Yes	Wang et al. (2019)

Labyrinthulomycetes have garnered widespread attention partially due to their ability to produce various high-value metabolites, including polyunsaturated fatty acids (PUFAs) and squalene. Currently, many studies are dedicated to screening high-yield fatty acid-producing thraustochytrid strains, optimizing cultivation conditions and breeding genetically engineered strains to increase the production of target compounds in thraustochytrids. However, studies on the isolation and fatty acid applications of *Labyrinthula* is severely limited. *Labyrinthula* sp. L72 isolated from the Seto Inland Sea, could not only efficiently utilize soy lecithin but also produce DHA (the only kind of PUFA in the cells), offering significant advantages for the industrial production of DHA (Kumon et al., 2005). Besides, *Labyrinthula* strains from the coastal waters of the South China Sea had been found to produce a large amount of PUFAs, with DHA accounting for more than 40% of the total fatty acids (Wang et al., 2019). The pathogen of SWD in *L. zosterae*, synthesizes DHA as its primary fatty acid on both artificial and algae-based media (Yoshioka et al., 2019). In addition, laboratory-inoculated and field-collected diseased eelgrass showed higher DHA content than controls, which suggested that *L. zosterae* might be an unrecognized natural source of PUFAs in the eelgrass ecosystem (Yoshioka et al., 2019). Our previous studies isolated 28 *Labyrinthula* strains evolutionarily close to thraustochytrids from the mangrove coastal areas of China, indicating a similar natural habitat and substrate selectivity between them (Wang et al., 2019). This result also suggested a potential coexistence and similar ecological functions between thraustochytrids and labyrinthulids (Wang et al., 2019). Recently, most studies on *Labyrinthula* focus on its pathogenicity toward seagrass, with fewer studies on metabolite composition analysis. Therefore, future studies on the metabolite analysis of *Labyrinthula* may provide new insights into revealing their interaction with seagrass in the natural habitats.

Seagrass diseases are an important cause of seagrass loss in some coastal areas. The protist *Labyrinthula* is an opportunistic pathogen of seagrass wasting disease (SWD) and is currently the focus of seagrass pathogen research (Table 2). In the early 20th century, 90% of the seagrass *Zostera marina* populations in the eastern United States and Western Europe died from this disease (Bergmann et al., 2011). High salinity, climate warming and eutrophication caused by human activities, escalate survival pressures on seagrass and diminish their adaptability. These factors are the primary risks contributing to the heightened frequency of seagrass disease outbreaks (Sullivan et al., 2018). Moreover, global changes may further facilitate the proliferation of pathogens. However, the specific mechanisms fueling the transmission of SWD remain elusive (Sullivan et al., 2013; Lee et al., 2021; Sullivan et al., 2023). Additionally, as saprophytic organisms, species of *Labyrinthula* are adept at decomposing organic materials, showcasing their potential in the bioremediation of coastal pollutants and playing a pivotal role in safeguarding marine ecosystems (Raikar et al., 2001). Hence, delving into the cultivable diversity, along with the physiological and biochemical properties, will not only shed light on their ecological significance but also bear crucial implications for the preservation of coastal environments and the advancement of marine aquaculture. To provide a comprehensive understanding of the protist *Labyrinthula* spp. and the seagrass disease caused by them, the taxonomy and ecological functions of *Labyrinthula* spp., as well as their relationship with seagrass disease under the influence of anthropogenic activities were summarized in this paper.

**Table 2:** Labyrinthulomycetes protists *Labyrinthula* causing disease of plants reported previously.

Strains	Habitat	Seagrass host	Sampling place	Sampling time	Disease symptom of plants	References
*Labyrinthula*	Marine	*Heterozostera nigricaulis*, *Zostera muelleri*	Southeastern Australian	March 2016 (beginning of the Australian autumn)	Black lesions in the leaf after infection for 3 days	Trevathan-Tackett et al. (2018)
*Labyrinthula* sp.	Marine	*Zostera muelleri*, *Halophila ovalis*, *Heterozostera nigricaulis,* and *Posidonia australis*	Southeastern Australian	From March 2014 to October 2015	–	Sullivan et al. (2017)
*Labyrinthula zosterae*	Marine	*Zostera marina*	Korea	From April to September 2013	Black lesions	Lee et al. (2021)
*Labyrinthula zosterae*	Marine	Eelgrass	Oregon, USA	In 2017 and 2019	–	Yoshioka et al. (2019)
*Labyrinthula zosterae*	Marine	*Zostera marina*	Northern European (Portugal, Germany, Denmark, southern Norway and western Sweden)	During 2010 to 2012	Black lesions, a sign of Labyrinthula-induced necrosis	Bockelmann et al. (2013)
*Labyrinthula zosterae*	Marine	*Zostera marina*	Northern Europe	Between August and October 2010	Black lesions in the leaf	Bockelmann et al. (2012)
*Labyrinthula* spp.	Terrestrial	Turfgrasses	New Mexico and Arizona	In 2011 and 2012	Rapid blight	Chitrampalam et al. (2015)
*Labyrinthula terrestris*	Terrestrial	Turfgrasses	USA	From 2003 to 2007	Rapid blight	Yadagiri et al. (2012)
*Labyrinthula terrestris*	Terrestrial	*Poa annua*	Colorado, USA	April 2009	Rapid blight	Hyder et al. (2010)
*Labyrinthula terrestris*	Terrestrial	Turfgrass *Poa trivialis* L. or *Lolium perenne* L.	Arizona, USA	February 2003	Rapid blight	Bigelow et al. (2005)

## 2 Taxonomy of *Labyrinthula* spp.

Labyrinthulomycetes have undergone several rearrangements, and the precise classification is unclear. However, recent revisions attempt to provide an updated higher-level taxonomic framework (Yokoyama et al., 2007; Pan et al., 2016; Doi and Honda, 2017). Distinguishing species within the genus *Labyrinthula* based on morphology and molecular biology continues to be a challenge. Combining ecological data with molecular phylogenetic data could play a crucial role in species delimitation within this group (Martin et al., 2016). Notably, only sequences of several *Labyrinthula* species have been described in GenBank, suggesting that the existing diversity of species and genera may be underestimated (Collado-Mercado et al., 2010; Martin et al., 2016; Adl et al., 2019). Furthermore, many newly isolated *Labyrinthula* strains cannot be assigned to specific species due to their as of yet, unresolved classification (Sullivan et al., 2017). Moreover, the lifestyle and ecological roles of *Labyrinthula* was unveiled, enriching the understanding of their physiological characteristics and ecological impacts (Dyková et al., 2008; Raghukumar and Damare, 2011; Burge et al., 2012). Therefore, further studies are necessary for a deeper understanding and accurate classification of the genus *Labyrinthula* and its members, which is crucial for revealing their roles and functions in natural habitats.

Analysis based on 18S rRNA has unveiled an extensive array of yet-to-be-described species within the genus *Labyrinthula*, noting that not all isolates result in necrotic lesions on seagrass (Sullivan et al., 2017). Research focusing on *Z. marina* illustrates that merely specific clades of isolates are implicated in disease manifestation, highlighting the genetic divergence between pathogenic and non-pathogenic strains of *Labyrinthula* (Bockelmann et al., 2012). Additional investigations, including geographical studies, toxicity assessments and sequencing of *Z. marina* in Europe, aimed to clarify the distinctions between pathogenic and non-pathogenic *Labyrinthula* (Brakel et al., 2014). The analysis of 170 recently isolated *Labyrinthula* strains sought to further identify their clades, host affiliations and geographical distribution (Martin et al., 2016). Moreover, *Labyrinthula* may become pathogenic under unfavorable environmental conditions (e.g., high water temperatures and insufficient light), while *Z. marina* seems to inhibit the growth of *Labyrinthula* by boosting its own growth and synthesizing phenolic acids (Brakel et al., 2014). Although the virulence of *Labyrinthula* exhibits variability, its pathogenicity remains high with a potential for cross-infecting hosts (Martin et al., 2016). This provides new criteria for the taxonomic study of *Labyrinthula* species. Collecting a broader array of samples illustrating interactions between seagrass and *Labyrinthula*, irrespective of the pathogenic status, opens novel avenues for elucidating species diversity of *Labyrinthula*, pathogenic mechanisms and intricate relationships with seagrass. This endeavor propels forward the research into its phylogeny and the evolutionary mechanisms underpinning its environmental adaptability (Martin et al., 2016).

## 3 Ecological functions of *Labyrinthula* spp.

*Labyrinthula* are mostly fungus-like heterotrophic protists that absorb nutrients through osmotrophic or phagotrophic means. Most *Labyrinthula* species are saprophytic, commonly associated with detritus such as fallen mangrove leaves, decaying algae and fecal pellets of marine invertebrates (Tsui et al., 2009). They also act as an opportunistic pathogen, linked to infections in marine algae (Sullivan et al., 2017) and terrestrial plants (Chitrampalam et al., 2015). The genus *Labyrinthula* is primarily associated with coastal environments. Since diatoms are major primary producers in coastal regions, interactions between *Labyrinthula* and diatoms could play a significant role in the decomposition of marine detritus (Popova et al., 2020; Tan et al., 2021). Specific strains isolated from surface marine sediments along the coasts of the Indian and Pacific Oceans, such as *L. diatomea*, are capable of utilizing marine diatoms *Cylindrotheca closterium* and *Micropodiscus weissflogii* as substrates for growth (Popova et al., 2020). Additionally, previous studies have shown that *Labyrinthula magnifica* and *L.* sp. also exhibit the capability to prey on diatoms (Grell, 1994). These diatom-associated *Labyrinthula* strains were colorless, but exhibited differences in dense clumps morphology, trophic cells and geographical distribution (Popova et al., 2020). These observations suggest potential species-level independence among them (Popova et al., 2020). While *Labyrinthula* has a global distribution, most known sequences originated from North America (Bai et al., 2019; Lee et al., 2021). This indicates that the diversity and ecological roles of *Labyrinthula* are still underestimated. In addition, *Labyrinthula* may consume different types of substrates or co-distribute with diatoms. Future studies on *Labyrinthula* and diatoms in natural habitats across different geographical ranges will help clarify the relationships within marine communities on a global scale.

Genomic sequencing of the pathogenic strain *L*. SR_Ha_C has shed new light on its role as a protistan pathogen and parasite, revealing a fascinating biological profile (Tan et al., 2021). The presence of genes associated with gliding motility and apical complex proteins, features typical of protistan pathogens, underscores its sophisticated mechanisms of movement and infection (Tan et al., 2021). Analysis of its carbohydrate-active enzymes (CAZymes) uncovered a diverse array of enzymes tasked with breaking down seagrass cell walls and managing carbohydrate reserves, highlighted by the identification of 112 genes linked to CAZy enzyme activity (Tan et al., 2021). These enzymes play a pivotal role in the marine carbon cycle and are likely crucial for the pathogen to breach living seagrass tissues via host adhesion (Tsui et al., 2009; Iwata and Honda, 2018). This discovery is significant for unveiling the molecular interaction mechanisms between host and pathogen, understanding the virulence of *Labyrinthula* and exploring its evolutionary mechanisms of ecological and environmental adaptability.

## 4 *Labyrinthula* spp. and the seagrass wasting diseases (SWD) under human activities

Environmental changes induced by extreme weather conditions and human activities severely impact seagrass ecosystems. These changes increase the mortality risk of seagrass, impair their carbon storage capabilities and adversely affect coastal ecological services (Orth et al., 2006; Graham et al., 2021). Increased precipitation due to strong storms and hurricanes facilitates a high load of organic nutrients and a decrease in pH levels, alongside adverse effects such as high temperatures and ocean acidification, leading to an increased mortality risk for seagrass in the future (Glibert et al., 2023). Urbanization and development increase estuarine runoff carrying sediments, nutrients and pollutants (pharmaceuticals, toxins, microplastics and pathogens), resulting in increased sediment loads in coastal waters, eutrophication, harmful algal blooms, fecal bacteria and a reduction in shellfish and fisheries (Freeman et al., 2019). These changes severely impact the seagrass bed ecosystems that provide essential ecological services, leading to a decrease in seagrass. Seagrass meadows contribute significantly to carbon storage and are an important sink for coastal blue carbon (Fourqurean et al., 2012; Serrano et al., 2021). Compared to seagrass beds less affected by human activities, carbon in seagrass beds more heavily influenced by human activities is more likely to escape when these systems are disturbed (Han et al., 2023). Thus, human activities have a definite impact on the carbon sequestration of seagrass bed ecosystems.

Aquaculture impacts the functionality associated with sediment bacteria in seagrass beds, particularly those functions related to carbon and nitrogen cycling (Sun et al., 2024). Eutrophication caused by aquaculture leads to a decline in seagrass abundance, affecting their carbon storage capacity (Han et al., 2024). The combined effects of shading and simulated grazing result in continuous loss of seagrass biomass, with significant increases in sulfide concentrations in sediment pore water and methane emissions from sediment surfaces (Lyimo et al., 2018). Human activities, such as dredging, expose coastal sediments to oxygen, disturbing the environment and reducing seagrass sediment carbon storage by promoting microbial remineralization (Macreadie et al., 2019). Thus, the reduction of seagrass due to human activities not only weakens their role in mitigating climate change, but may also promote the release of significant amounts of greenhouse gases into the environment from seagrass meadows (Lyimo et al., 2018). Moreover, with increasing nutrient loads, the biomass of sediment microbes and extracellular enzyme activity in seagrass beds increase, stimulating the remineralization of sediment organic carbon and potentially altering seagrass blue carbon (Liu et al., 2017). Furthermore, seagrass meadow bacteria can enhance the removal of pathogens by promoting particle settling (Liu et al., 2023). But the reduction and fragmentation of seagrass meadows due to human activities and global climate change inhibit the assumed potential for bacterial pathogen removal (Liu et al., 2023). On the other hand, stress stimuli from human activities may induce seagrass to release antibacterial compounds, inhibiting pathogens and thereby promoting the clearance of assumed bacterial pathogens in seagrass meadows (Deng et al., 2021). Therefore, environmental changes caused by human activities have significant impacts on both seagrass microbial communities and pathogens, potentially promoting the outbreak and development of seagrass diseases. One such concerning disease is the wasting disease of seagrass caused by the protist *Labyrinthula*, a member of the class Labyrinthulomycetes.

### 4.1 The occurrence of seagrass diseases

**Figure 1:**
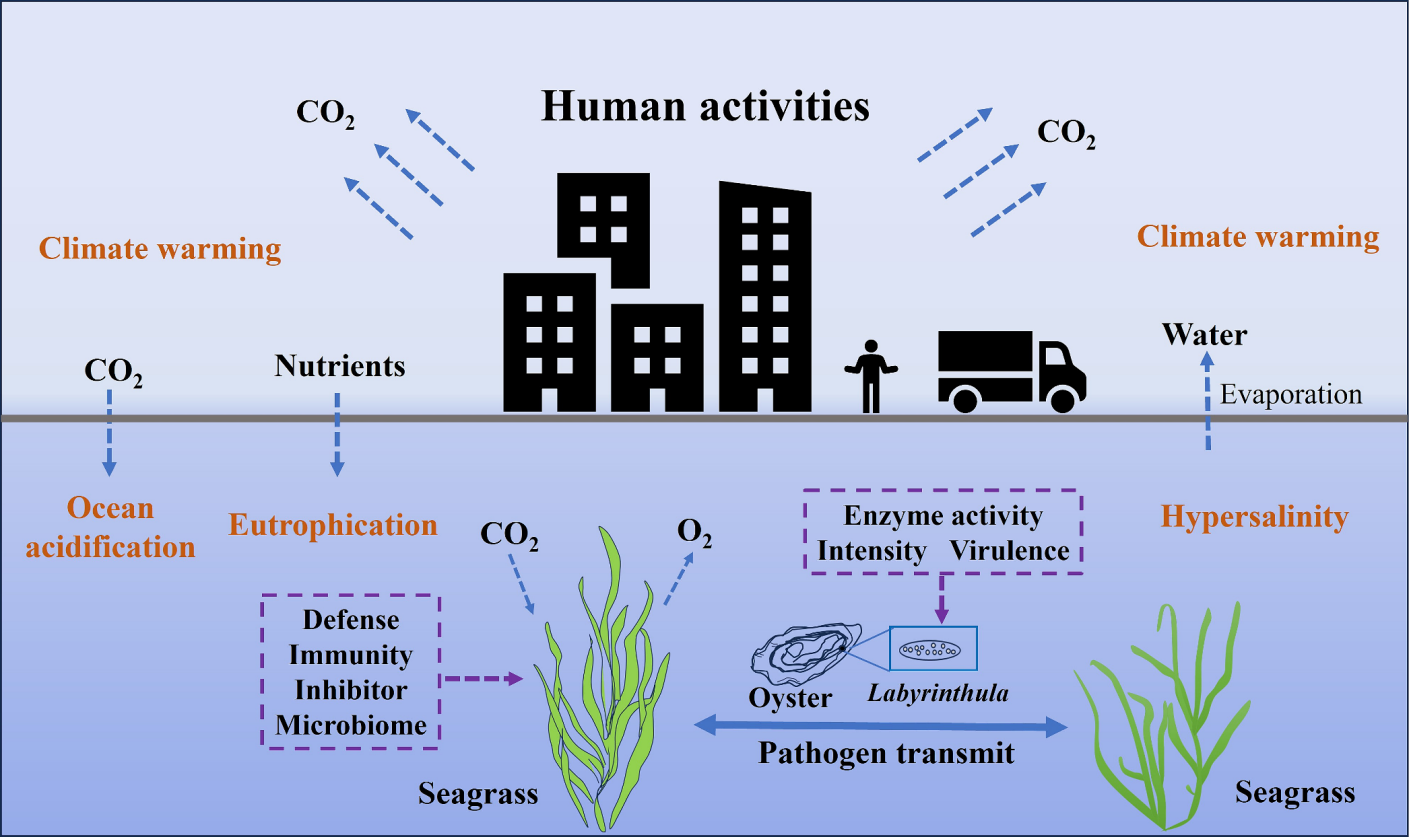
The relationship between the opportunistic pathogen *Labyrinthula* and the seagrass host under the influence of human activities.

The pathogenic mechanisms of *Labyrinthula* sp. and the defense strategies of seagrass are crucial for understanding the occurrence and control of seagrass diseases. Currently, conditions leading directly to seagrass disease outbreaks remain contentious. The disease outbreaks not only depend on the health status and defensive capabilities of the seagrass, but also closely relate to the virulence and enzymatic capabilities of *Labyrinthula* strains (Figure 1). For instance, metabolites from the tropical seagrass *Thalassia testudinum*, including phenolic and non-phenolic compounds, have been proven to inhibit the growth of pathogenic *Labyrinthula* sp. (Trevathan-Tackett et al., 2015). This suggests seagrass defend against wasting disease by producing specific metabolites. Moreover, laboratory infection trials have revealed variations in virulence among different *Labyrinthula* strains, with highly virulent strains causing typical black leaf lesions in seagrass leaves (Bockelmann et al., 2012; Brakel et al., 2014; Martin et al., 2016). To further investigate this phenomenon, two techniques were developed, including a qPCR method for quantitatively assessing pathogen load and an immunolabeling method for measuring immune response of the host (Duffin et al., 2020). Both of these methods demonstrated high sensitivity and positive correlation with biomarkers related to abiotic stress (Duffin et al., 2020). These findings highlight the complexity of the interaction mechanisms between seagrass and *Labyrinthula*, necessitating a comprehensive study on seagrass disease prevention and control strategies.

### 4.2 Response of *Labyrinthula* and the seagrass host to environmental variations

Environmental stressors such as increased salinity and climate warming are significant factors influencing the susceptibility of seagrass to diseases (Table 3). Elevated salinity has been shown to cause a significant rise in H_2_O_2_ concentration within the seagrass *T. testudinum*, surpassing the growth inhibition concentration for external *Labyrinthula* (Trevathan et al., 2011). However, due to the direct effect of osmotic pressure on pathogens and the indirect effect of host-generated reactive oxygen species, short-term exposure to high salinity does not necessarily lead to an immediate increase in susceptibility to wasting disease (Trevathan et al., 2011). Temperature is also a critical factor affecting the prevalence of wasting diseases in seagrass. Field investigations correlated long-term satellite remote sensing of ocean temperature data with field survey results from 32 seagrass meadows along the North American Pacific coast (Aoki et al., 2022). This study revealed that the risk of wasting disease, caused by the protist *Labyrinthula*, increased with the rising temperatures (Aoki et al., 2022). Similarly, models enhanced with machine learning algorithms and field survey results suggested that disease prevalence and severity decreased under cooler conditions, and diseases were suppressed in healthier and denser meadows (Graham et al., 2023). Field experiments, conducted across a wide geographic area and from a macro perspective, have established the relationship between climate warming and seagrass diseases. However, these often lack detailed attention to seagrass, and fail to reveal the specific mechanisms through which warming influences seagrass diseases, as well as the resistance capabilities and corresponding mechanisms of seagrass.

**Table 3:** Impacts of environmental changes on *Labyrinthula* and the seagrass host.

*Labyrinthula* pathogen	Host	Environmental change	Experimental method	Response of *Labyrinthula*	Response of host	References
*Labyrinthula* sp.	*Thalassia testudinum* Banks ex König	Short-term hypersalinity exposure	Laboratory experiment with seagrass exposed to ambient (30 psu) and elevated (45 psu) salinities before infected for 1 week by *Labyrinthula* sp.	750 μM H_2_O_2_ (over 9 times the concentration that was shown to inhibit *Labyrinthula* colony growth *in vitro*) *in vivo*	Reduced infection intensity, no reduced prevalence	Trevathan et al. (2011)
*Labyrinthula* sp.	Climate vulnerable Mediterranean seagrass, *Posidonia oceanica*	Warming	Laboratory experiments from 24°C to 32°C	Inhibited cell division and growth under above 28°C	Reduced risk of wasting disease, relieve pathogen pressure	Olsen et al. (2014)
*Labyrinthula zosterae*	*Zostera marina*	Climate warming	Field study combining long-term satellite remote sensing of ocean temperature with field surveys		Increase risk of wasting disease	Aoki et al. (2022)
*Labyrinthula zosterae*	*Zostera marina*	Warming	Eight-week warming experiment in the laboratory	Slightly increased intensity of *Labyrinthula*	Reduced infection intensity with no reduced prevalence	Schenck et al. (2023)
*Labyrinthula zosterae*	Eelgrass	Deeper habitats and cooler temperatures	Field surveys assisted with a machine-language algorithm		Reduced disease prevalence and severity under cooler conditions, suppressed disease in healthy and higher density meadows	Graham et al. (2023)
*Labyrinthula* sp.	Plastic waste	Ocean acidification	A mesocosm experiment in the laboratory	Obviously affected by the increase of CO_2_		Zhang et al. (2023)
*Labyrinthula* sp.	Pacific oyster spat (*Magallana gigas*)	Ocean warming and acidification	A fully crossed designs with different temperatures (18 and 24°C) and CO_2_ levels (800, 1,600 and 2,800 μatm)	Differed in abundance between temperatures at 18°C, No differentially abundant taxa among the three CO_2_ concentration conditions		Zhong et al. (2024)

Laboratory experiments offer a feasible pathway for detailing the environmental responses of seagrass and pathogens. Yet related studies are currently limited. The impact of temperature on disease is non-linear for most pathogen-host relationships. However, laboratory experiments indicated that warming may reduce the risk of wasting disease in the seagrass *Posidonia oceanica* and alleviate pathogenic pressure from this species (Olsen et al., 2014). Besides, laboratory simulations of warming effects on seagrass diseases have found that seagrass hosts with diverse genotypes reduce infection intensity, compared to hosts with less diversity (Schenck et al., 2023). However, since seagrass genotype diversity actually slightly increased the intensity of *Labyrinthula* under environmental temperatures, the prevalence of seagrass diseases did not decrease (Schenck et al., 2023). Therefore, more systematic and in-depth research is needed to reveal the specific seagrass characteristics and environmental conditions of the study area. Additionally, ocean acidification has a strong impact on plastisphere communities within mesocosm ecosystems, and *Labyrinthula* is also significantly affected by increased CO_2_ (Zhang et al., 2023). However, when analyzing the cross-effects of temperature (18°C and 24°C) and CO_2_ concentrations (800, 1,600 and 2,800 μatm) on *Labyrinthula*, it was found that the density of *Labyrinthula* varied at different temperatures (Zhong et al., 2024). And there was little difference under the three CO_2_ concentration conditions (Zhong et al., 2024). Thus, ocean warming has a more significant impact on *Labyrinthula* (Zhong et al., 2024). Additionally, previous studies had demonstrated that various environmental stressors significantly affect seagrass health (Lefcheck et al., 2017; Hensel et al., 2023). The interaction and response mechanisms between seagrass hosts and *Labyrinthula* pathogens become more complex under the combined effects of climate warming, ocean acidification, emerging pollutants, typhoons and other factors. Therefore, establishing comprehensive multi-factor prediction models in the future will provide more useful information for the conservation and restoration of seagrass beds.

### 4.3 Interactions between *Labyrinthula* spp. and the seagrass host

In addition to environmental factors that have a significant impact on the occurrence and development of SWD, the immune status of seagrass also plays a crucial role in the interaction between seagrass and *Labyrinthula*, as well as in the outbreaks of seagrass wasting disease (Table 4). The pathogen *Labyrinthula* could promote its growth and adapt to various environmental stresses by modulating the expression of seagrass defense genes, particularly by upregulating genes related to phenol synthesis (Brakel et al., 2014; Duffin et al., 2021; Venkataraman et al., 2023). This strategy can enhance immunity even during periods of scarce resources, although it may fail under extreme physiological challenges (Duffin et al., 2021). The interaction between *Labyrinthula* and seagrass, through the regulation of seagrass defense gene expression and immune responses, could adapt to environmental pressures and resource availability, promoting seagrass growth and regulating pathogen load under certain conditions (Duffin et al., 2021). Furthermore, seagrass-associated microbes and their metabolic products have some inhibitory activity against seagrass pathogens (Ugarelli et al., 2024). Meanwhile, metabolites from the natural microbiome of seagrass may provide food for *Labyrinthula* and thus promote its growth (Duffin et al., 2021). However, when seagrass are inoculated with *Labyrinthula* after artificially killing the natural microbiome, the severity of seagrass disease significantly reduced (Graham et al., 2024). This might indicate that environmental stress conditions such as extreme temperatures, ocean acidification and substantial discharges of coastal pollutants could reduce the outbreaks of SWD and lessen its severity (Graham et al., 2024). Additionally, laboratory experiments have shown that marine organisms could serve as effective sinks and potential sources of seagrass pathogens. The presence of oysters could significantly reduce the severity and infection intensity of SWD (Agnew et al., 2022). But oysters previously exposed to *L. zosterae* could also transmit the pathogen to local eelgrass (Agnew et al., 2022). Whether oysters transmit *Labyrinthula* under field conditions remains unclear (Agnew et al., 2022). Therefore, future research on the interaction between seagrass and *Labyrinthula*, incorporating the cross-effects of coexisting marine organisms and environmental factors, will provide richer and more critical information for the prevention and treatment of seagrass diseases.

**Table 4:** Studies on the interaction between *Labyrinthula* pathogen and the seagrass host.

*Labyrinthula* pathogen	Host	Experimental method	Interaction	References
*Labyrinthula zosterae* (Lz)	European *Zostera marina*	Genotype × genotype interactions of host and pathogen from different regions (10 ~ 100 km-scale) through reciprocal infection in the laboratory	Decreased mean Lz abundances below 10%. Enhanced leaf growth, no increase in mortality, strong immune modulation of the host defense by a potential parasite, one strongly up-regulated gene involved in phenol synthesis	Brakel et al. (2014)
*Labyrinthula* spp.	*Thalassia testudinum*	Assessing the activity of four immune biomarkers in conjunction with pathogen prevalence and load [via quantitative PCR (qPCR)] at 15 sites across Florida Bay	Resistance strategies to up-regulate the immunity of plants in places with scarce resources, a high pathogen load under physically challenging conditions	Duffin et al. (2021)
*Labyrinthula zosterae* (Lz)	Zostera marina	Two temperature (11°C or 18°C) treatments and acclimation for 10 days, and exposion to a waterborne inoculation containing infectious Lz or sterile seawater	A limited repertoire of virulence approaches in Lz, including degradation of cell walls, consumption of intracellular materials and movement within the host. A wide range of responses to infection in the eelgrass, from cascading phytohormone signals to altered production of phenols, increased PAMP/DAMP signaling and apoptosis	Venkataraman et al. (2023)
*Labyrinthula* spp.	*Thalassia testudinum*	Leaf mycobiome sequencing, organic compounds isolation and extraction from endophytes to test their anti-*Labyrinthula* potential using disk diffusion assays	Strong inhibition of two fungal endophytes isolates. Confirmed bioactivity of Cytosporone B from *D*. sp. M14 against *Labyrinthula*	Ugarelli et al. (2024)
*Labyrinthula zosterae*	*Zostera marina*	Experimentally reduced eelgrass microbiome with antibiotics and bleach, then inoculated with *Labyrinthula zosterae*	Improved growth of *Labyrinthula* by obtaining microbial metabolites as food. Significantly less disease severity of eelgrass	Graham et al. (2024)
*Labyrinthula zosterae*	*Zostera marina*	Laboratory experiment with Pacific oyster *Crassostrea gigas* and eelgrass exposure to *Labyrinthula zosterae*, field experiment with eelgrass ramets deployed with and without oysters adjacent to eelgrass known to have SWD	*C. gigas* acting as an effective sink and a source of *Labyrinthula zosterae* in the laboratory study	Agnew et al. (2022)

## 5 Conclusion

The effects of environmental stresses exacerbated by human activities and global climate changes on seagrass health highlight the complex interactions between *Labyrinthula* and its host seagrass. This review summerizes the ecological function, pathogenicity and role in seagrass ecosystems of the Labyrinthulomycetes protist *Labyrinthula*. As an opportunistic pathogen, *Labyrinthula* spp. pose a significant threat to global seagrass beds by causing SWD. Despite progress in phylogenetic analysis, understanding pathogenic mechanisms and studies on environmental adaptability, information on its biogeography, host specificity and ecological functions remains scarce. Moreover, though the potential for PUFAs synthesis of *Labyrinthula* is beginning to be recognized, studies on its metabolites are still in its infancy. Future research needs to explore the interactions between *Labyrinthula* and seagrass, as well as other natural hosts more deeply and across a broader geographical range, including how these interactions are affected by environmental factors. Specifically, there is a need to strengthen research on the adaptability mechanisms of *Labyrinthula* under varying environmental conditions, including its response to global climate change. Additionally, increasing research efforts into the species diversity, distribution and ecological roles of *Labyrinthula* is crucial for understanding its role in particular these areas where human activity is frequent.

## Author contributions

QW: Conceptualization, Funding acquisition, Project administration, Writing – original draft, Writing – review & editing. XY: Writing – original draft. YH: Writing – review & editing. YZ: Writing – original draft, Writing – review & editing. RH: Writing – original draft. HY: Writing – original draft. CW: Writing – original draft. MB: Conceptualization, Writing – review & editing.
